# Synthesis, Antimicrobial Activity and Molecular Docking of Novel Thiourea Derivatives Tagged with Thiadiazole, Imidazole and Triazine Moieties as Potential DNA Gyrase and Topoisomerase IV Inhibitors

**DOI:** 10.3390/molecules25122766

**Published:** 2020-06-15

**Authors:** Heba E. Hashem, Abd El-Galil E. Amr, Eman S. Nossier, Elsayed A. Elsayed, Eman M. Azmy

**Affiliations:** 1 Department of Chemistry, Faculty of Women, Ain Shams University, Heliopolis, Cairo 11457, Egypt; hebahashem89@yahoo.com (H.E.H.); eman.azmy@women.asu.edu.eg (E.M.A.); 2 Drug Exploration & Development Chair (DEDC), Pharmaceutical Chemistry Department, College of Pharmacy, King Saud University, Riyadh 11451, Saudi Arabia; 3 Applied Organic Chemistry Department, National Research Center, Dokki 12622, Cairo, Egypt; 4 Pharmaceutical Medicinal Chemistry Department, Faculty of Pharmacy (Girls), Al-Azhar University, Cairo 11754, Egypt; dr.emannossier@gmail.com; 5 Bioproducts Research Chair, Zoology Department, Faculty of Science, King Saud University, Riyadh 11451, Saudi Arabia; eaelsayed@ksu.edu.sa; 6 Chemistry of Natural and Microbial Products Department, National Research Centre, Dokki 12622, Cairo, Egypt

**Keywords:** thiourea, antimicrobial, *E. coli* DNA B gyrase, *E. coli* Topoisomerase IV, molecular docking

## Abstract

To develop new antimicrobial agents, a series of novel thiourea derivatives incorporated with different moieties **2–13** was designed and synthesized and their biological activities were evaluated. Compounds **7a**, **7b** and **8** exhibited excellent antimicrobial activity against all Gram-positive and Gram-negative bacteria, and the fungal *Aspergillus flavus* with minimum inhibitory concentration (MIC) values ranged from 0.95 ± 0.22 to 3.25 ± 1.00 μg/mL. Furthermore, cytotoxicity studies against MCF-7 cells revealed that compounds **7a** and **7b** were the most potent with IC_50_ values of 10.17 ± 0.65 and 11.59 ± 0.59 μM, respectively. On the other hand, the tested compounds were less toxic against normal kidney epithelial cell lines (Vero cells). The in vitro enzyme inhibition assay of **8** displayed excellent inhibitory activity against *Escherichia coli* DNA B gyrase and moderate one against *E. coli* Topoisomerase IV (IC_50_ = 0.33 ± 1.25 and 19.72 ± 1.00 µM, respectively) in comparison with novobiocin (IC_50_ values 0.28 ± 1.45 and 10.65 ± 1.02 µM, respectively). Finally, the molecular docking was done to position compound **8** into the *E. coli* DNA B and Topoisomerase IV active pockets to explore the probable binding conformation. In summary, compound **8** may serve as a potential dual *E. coli* DNA B and Topoisomerase IV inhibitor.

## 1. Introduction

It is widely known that there is a great demand for discovery of new antibacterial compounds due to the rising and global problem of antibiotic resistance [1]. Searches for new compounds via screening against specific molecular targets have applied to furnish lead compounds for antibiotic development [2].

Thiourea and thiosemicarbazide are two sulfur-bearing scaffolds, which are present in the many biologically active agents with antibacterial, antifungal, antioxidant, antitumor and anticonvulsant activities [3,4,5,6,7]. Thiourea derivatives act as precursors for the synthesis of various classes of acyclic and heterocyclic compounds, in addition to their high biological activity [8]. Moreover, Thiosemicarbazides are not only intermediate compounds for the synthesis of various bioactive heterocycles such as pyrazole thiazole, thiadiazole, triazole, triazepine, oxadiazole, thiadiazine, thiadiazepine and tetrazole [9,10,11] but also has been useful for the design of biologically active agents and could assist as linkers between efficient moieties providing lengths sufficient for nice embedding in the vital receptors. These targets exhibited antiviral, antiamebal, antifungal, antimalarial, antiproliferative and antinociceptive activities [12]. They are also widely used in the treatment of different microbial infections especially p-acetamidobenzaldehyde thiosemicarbazone (thiacetazone) that has been utilized for more than 50 years against *Mycobacterium tuberculosis* [13].

**Figure 1:**
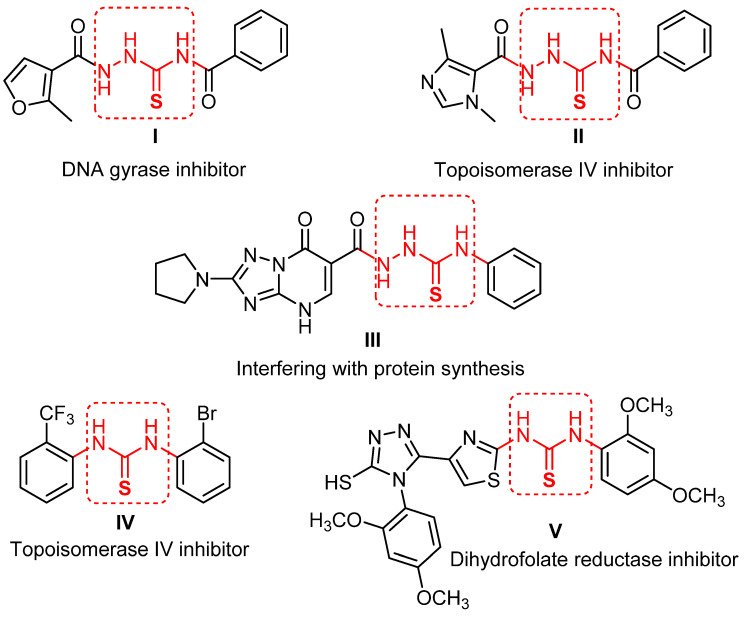
Recently discovered thiourea and thiosemicarbazide derivatives having antimicrobial activities via different mechanisms of action.

Recently in the search for novel antimicrobial agents, it was found that the reported thiosemicarbazide I significantly inhibits the activity of *Staphylococcus aureus* DNA gyrase with IC_50_ value of 14.59 μM [14]. The replacement of furane moiety in I with imidazole one in 4-benzoyl-1-(4-methyl-imidazol-5-yl)carbonylthiosemicarbazide (II) represents inhibitory activity against topoisomerase IV but not against DNA gyrase [15]. However, 2-pyrrolidin-4,7-dihydro-7-oxo-1,2,4-triazolo [1,5-*a*]pyrimidine (III) increased the activity against both Gram positive and Gram negative bacteria through interfering with protein synthesis [16]. *N*-arylthiourea IV explored the highest potency against standard and methicillin-resistant *Staphylococcus aureus* and *Staphylococcus epidermidis* strains through its inhibitory effect on topoisomerase IV [17]. The thiourea V was proved to be 2.7 fold more active than the positive control methotrexate as a dihydrofolate reductase (DHFR) inhibitor [18] (Figure 1).

Based on the above observations, structures involved in Figure 1 and as part of our ongoing program aimed at the discovery and development of new antimicrobial targets [19,20,21,22,23,24,25,26], in this work, a series of novel thiourea and thiosemicarbazide derivatives bearing different moieties 2–13 were designed by similarity and synthesized to be topoisomerase inhibitors. Encouraged by the fact that thiourea and thiosemicarbazide derivatives are reported to exhibit various potential antimicrobial activities, i.e., kinases, as previously described, we aimed to evaluate newly synthesized derivatives in terms of their possible antimicrobial as well as anticancer potentials. Furthermore, the mechanism of action of these new derivatives will be investigated for their inhibitory effects against three kinases, *E. coli* DNA gyrase B, *E. coli* Topoisomerase IV and dihydrofolate reductase. Finally, molecular docking was done to prove the mechanism of action and determine the essential structural features responsible for the antimicrobial efficacy. 

## 2. Results and Discussion

### 2.1. Chemistry

**Scheme 1:**
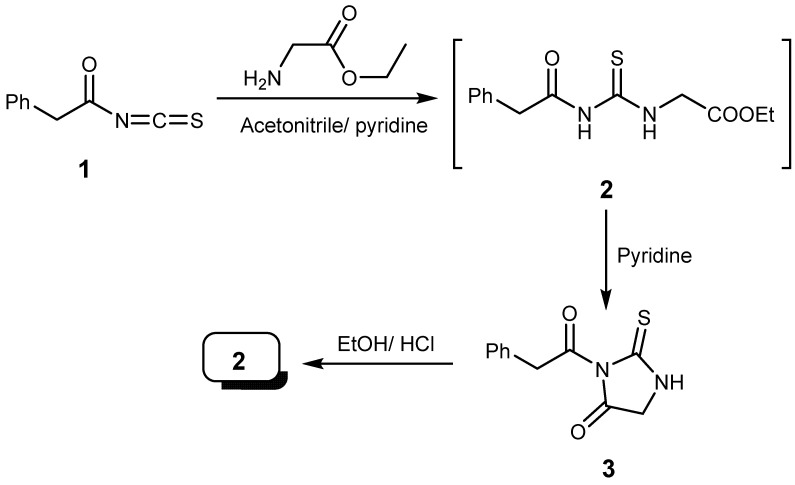
Synthetic route for compounds **2** and **3**.

Reaction of benzylisothiocyanate **1** and ethyl glycinate in the presence of a small amount of pyridine gave thiourea derivative **2** as an intermediate, which was cyclized in situ to 3-(2-phenyl-acetyl)-2-thioxoimidazolidin-4-one (**3**), which was opening by refluxing in ethanol/hydrochloric acid to obtain thiourea derivative **2** (Scheme 1). The ^1^HNMR for the linear-adduct **2** revealed the presence of two singlet signals for NH protons in the downfield region, as well as triplet and quartet signals for the ethyl group (CH_3_CH_2_) beside two singlet signals at δ 3.46 and δ 3.79 for 2CH_2_ protons and a multiplet signals for phenyl protons. On the other hand, IR spectrum of **3** shows high absorption band of cyclic carbonyl group at 1741 cm^−1^ and its ^1^HNMR spectrum displays a broad singlet signal for the NH proton that is exchangeable with D_2_O.

**Scheme 2:**
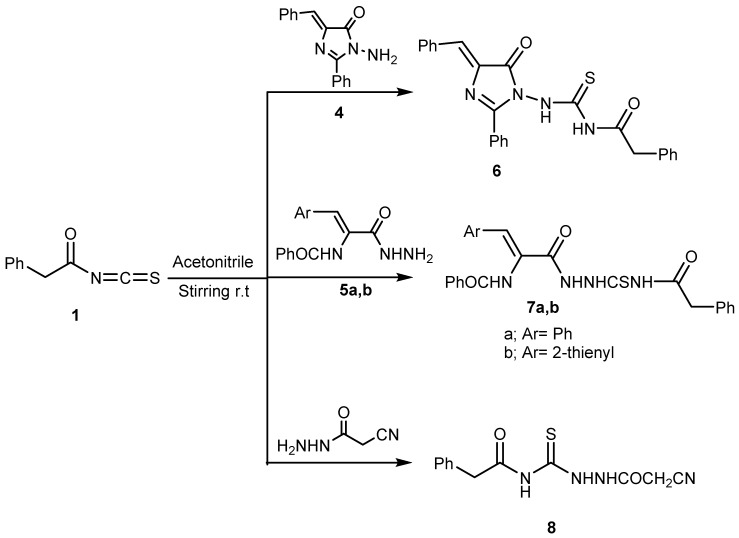
Synthetic route for thiosemicarbazides **6**–**8.**

Treatment of isothiocyanate **1** with N-amino imidazole derivative **4** [27], carbohydrazide derivative **5a,b** [28] or cyanoacetohydrazide in acetonitrile at room temperature with stirring afforded the corresponding 1-(4-benzylidene-4,5-dihydro-5-oxo-2-phenylimidazol-1-yl)-3-(2-phenylacetyl)thiourea (**6**), and thiosemicarbazide derivatives **7a,b** and **8**, respectively (Scheme 2).

**Scheme 3:**
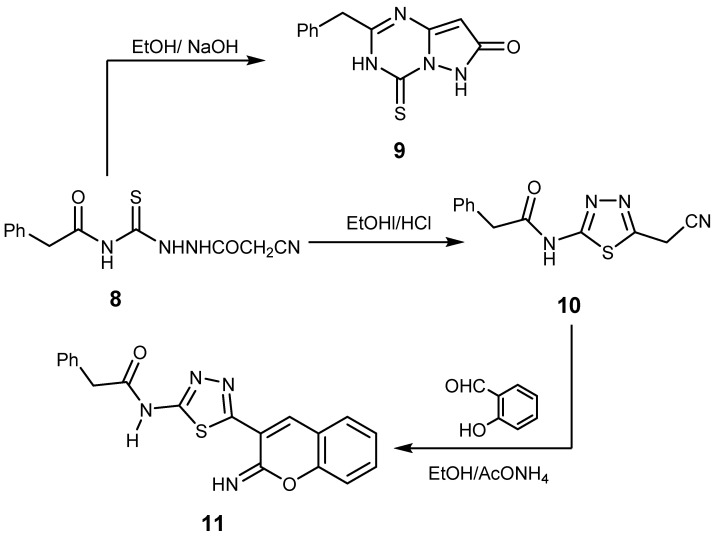
Synthetic route for derivatives **9**–**11**.

Cyclization of thiosemicarbazide derivative **8** by heating in ethanol, in the presence of sodium hydroxide or hydrochloric acid afforded the corresponding pyrazolotriazinone derivative **9** and N-(5-(cyanomethyl)-1,3,4-thiadiazol-2-yl)-2-phenylacetamide **10**, respectively. The latter compound **10** was reacted with salicaldehyde in refluxing ethanol, in the presence of ammonium acetate to give the thiadiazolochromen derivative **11** (Scheme 3).

**Scheme 4:**
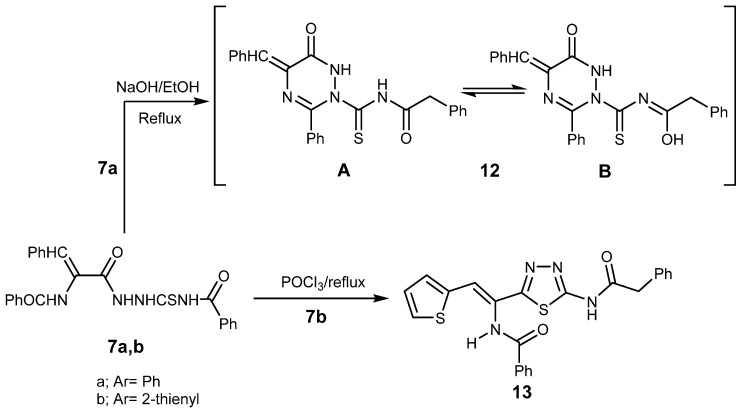
Synthetic route for derivatives **12** and **13**.

Finally, cyclization of **7a** with ethanolic sodium hydroxide or **7b** with phosphorus oxychloride afforded the corresponding cyclized products, 1,2,4-triazinone derivative **12** and thiadiazole derivative **13**, respectively (Scheme 4). The structure of **12** was elucidated from its spectral data, IR spectrum showed absorption band correlated with C=O, C=N and C=S groups. The ^1^HNMR spectrum of compound **12,** which shows from low to high field the absorption of exchangeable with D_2_O, aromatic and aliphatic protons is in a good agreement with the proposed structure. Inspection of the ^1^HNMR spectrum revealed the existence of compound **12** in dimethylsulphoxide solution as an equilibrium mixture of tautomers **12A** and **12B**, because the presence of extra broad singlet at δ 13.64 due to the presence of OH (structure **12B**).

### 2.2. Biological Activity

#### 2.2.1. Antimicrobial Sensitivity Assay

**Figure 2:**
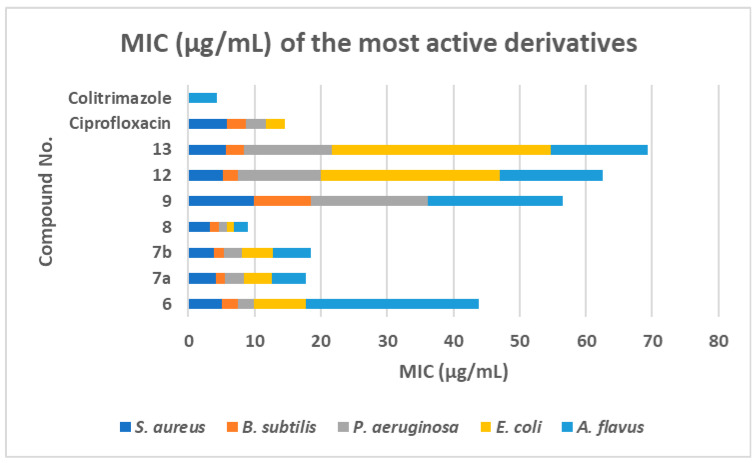
Antimicrobial activity (MIC) of the most active compounds against different bacterial and fungal *A. flavus* strains compared with the reference drugs, ciprofloxacin and clotrimazole, respectively.

The antimicrobial activity of all synthesized compounds **2**–**13** was screened against a panel of two Gram-positive bacteria (*Staphylococcus aureus* ATCC 29213 and *Bacillus subtilis* ATCC 6633), two Gram-negative bacteria (*Escherichia coli* ATCC 25922, *Pseudomonas aeruginosa* ATCC 27853) and two fungi (*Candida albicans* ATCC 10231 and *Aspergillus flavus* ATCC 46283) using the agar well diffusion method [24]. Ciprofloxacin and clotrimazole were used as antibacterial and antifungal standards, respectively. The results of this study were recorded as the average diameter of the inhibition zones (IZ) in Table 1. It was found that compounds **7a**, **7b** and **8** revealed excellent improved antimicrobial activity against all Gram-positive and Gram-negative bacteria, and the fungal *A. flavus* while moderate activity against *C. albicans*. Moreover, compound **6** displayed higher activities against Gram positive bacteria (*S. aureus* and *B. subtilis*) and the Gram-negative *P. aeruginosa* and weak activity against the remaining strains. Additionally, the promising activity against *S. aureus* was observed from compounds **9**, **12** and **13**, however against *B. subtilis* from compounds **12** and **13**. The remaining derivatives demonstrated potencies from moderate to weak in comparison with the reference drugs. The antifungal potency against *C. albicans* for all tested derivatives ranged from weak to no activity at all. Additionally, it was noticed that compounds **2** and **3** showed no activity against all the screened strains. The most active targets **6, 7a, 7b, 8, 9, 12** and **13** were further investigated for the assignment of the minimum inhibitory concentration (MIC) (Table 2, Figure 2). Compound **8** explored the best potential MIC values ranged from 0.95 ± 0.22 to 3.25 ± 1.00 μg/mL in comparison with that of the standard compounds, followed by **6**, **7a**, **7b**, **12** and **13** (MIC ranged from 1.39 ± 0.50 to 33 ± 0.10 μg/mL).

##### Structure–Activity Relationship (SAR)

Regarding to the structure–activity relationship (SAR) study, it was noted that the attachment of phenyl acetyl fragment to thiourea and ethyl acetate groups in compound **2** or to thioxoimidazolidine moiety in **3** abolished the antimicrobial activity against almost tested strains. Replacement of ethyl acetate in **2** with cyanoacetamide in compound **8** explored the highest antimicrobial activity against all strains except *C. albicans*. By fixation of thiourea bearing phenyl acetyl group, insertion of 4-benzylidene-5-oxo-2-phenylimidazole on the other side in **6** displayed excellent activity against *S. aureus*, *B. subtilis* and *P. aeruginosa* with MIC values of 5.12 ± 0.05, 2.29 ± 0.05 and 2.48 ± 0.11 μg/mL, respectively. While, insertion of 2-benzamido-3-(phenyl or thienyl)-acryloyl fraction in **7a** and **7b**, exhibited superior antimicrobial activity against all screened strains (MIC ranged from 1.39 ± 0.50 to 5.70 ± 0.01 μg/mL). Cyclization of the thiosemicarbazide **8** to pyrazolotriazinone **9**, 5-cyanomethyl-1,3,4-thiadiazole **10** or thiadiazole chromen **11** dropped the antimicrobial activity. On the other hand, cyclization of **7a** and **7b** to 2-phenylethylidene-6-oxo-3-phenyl-1,2,4-triazine **12** and 1,3,4-thiadiazol-2-yl-2-thien-2-yl-vinyl-benzamide **13**, respectively retained the potency against the Gram positive strains (*S. aureus* and *B. subtilis*; MIC values of 5.22 ± 0.03 and 5.60 ± 0.15 μg/mL for *S. aureus* and 2.30 ± 0.05 and 2.72 ± 1.11 μg/mL for *B. subtilis*, respectively) with remarked drop in the activity against the remaining strains.

#### 2.2.2. Cytotoxic Activity Using MTT Assay 

The cytotoxic activities of the highly active compounds as antimicrobials **6, 7a, 7b, 8, 9, 12** and **13** were evaluated against human breast cancer (MCF-7) as well as normal kidney epithelial cell line (Vero) cells using the MTT method [24] and cisplatin as a reference. The results are expressed as IC_50_ values (µM) and are depicted in Table 3. It can be seen that the order of potential cytotoxicity can be arranged as **7a** > **7b** > **8** > **6** > **9** > **12** > **13**. Compounds **7a** and **7b** were the most potent showing the least obtained IC_50_ values of 10.17 ± 0.65 and 11.59 ± 0.59 μM, respectively. These values were more or less comparable to the tested positive control (cisplatin: IC50 8.89 ± 0.37 μM). On the other hand, the tested compounds were less toxic against normal kidney epithelial cell lines (Vero cells).

The data obtained from antimicrobial and cytotoxic screening revealed that compounds **7a, 7b** and **8** displayed the highest potency as antimicrobial with low toxicity.

#### 2.2.3. In Vitro Enzyme Assay

In order to validate the mode of action of the highly potent compound **8** as antimicrobial, it was assessed for in vitro inhibition against three kinases, *E. coli* DNA gyrase B, *E. coli* Topoisomerase IV and dihydrofolate reductase (DHFR) using the reported procedures [22,24,29]. This test was performed at the Confirmatory Diagnostic Unit, VACSERA, Egypt. Novobiocin was used as a standard reference for *E. coli* DNA gyrase B and *E. coli* Topoisomerase IV, while methotrexate as the standard for DHFR. The results were recorded as IC_50_ values in μM and listed in Table 4.

As expressed in Table 4, compounds **8** exhibited excellent inhibitory activity against *E. coli* DNA B gyrase in comparison with novobiocin (IC_50_ = 0.33 ± 1.25 and 0.28 ± 1.45 µM, respectively). Moreover, it showed moderate inhibitory potency against *E. coli* Topoisomerase IV, about half the potency of novobiocin (IC_50_ values 19.72 ± 1.00 and 10.65 ± 1.02 µM, respectively). On the other hand, compound **8** cannot be considered as the DHFR inhibitor by comparing its IC_50_ value with that of methotrexate (IC_50_ values 189.47 ± 1.06 and 0.14 ± 1.62 µM, respectively). 

### 2.3. Molecular Modeling Study

Prompted by the kinase inhibitory results, compound **8** was selected for molecular docking against *E. coli* DNA gyrase B and Topoisomerase IV using MOE (Molecular Operating Environment) software 10.2008 [30]. The protein data bank files (PDB: 1AJ6 and 1S14) [26,31] was downloaded and the docking simulation was verified firstly by redocking of the native ligand (novobiocin) in the binding pockets of *E. coli* DNA gyrase B and Topoisomerase IV with energy score (S) = −10.77 and −7.88 kcal/mol and root mean standard deviation (RMSD) = 0.86 and 0.79 Å, respectively.

**Figure 3:**
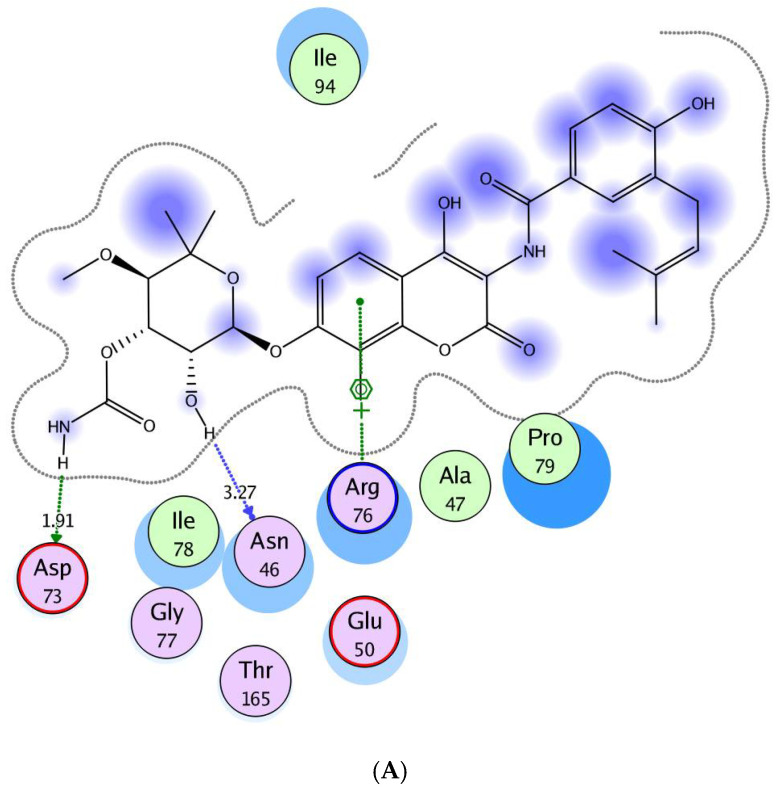

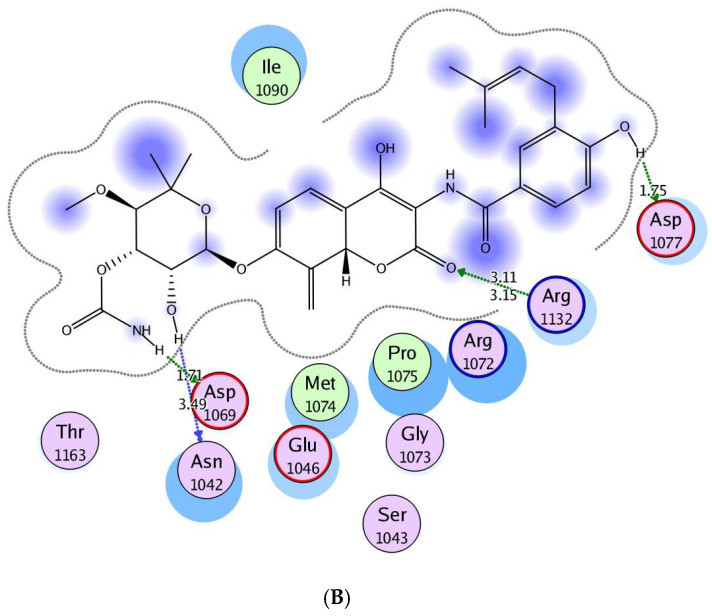
Two-dimensional interaction diagrams of novobiocin redocked in the active sites of *E. coli* DNA gyrase B (PDB ID: 1AJ6) (**A**) and Topoisomerase IV (PDB ID: 1S14) (**B**), respectively using Molecular Operating Environment (MOE) software. H-bond interactions with the proteins are shown as dashed arrows.

As reported previously in docking of novobiocin [26,31], fixation within the active site of DNA gyrase B kinase was done through two hydrogen bonds with the essential amino acids **Asp46** and **Asp73** and arene-cation interaction with **Arg76**. While within the ATP binding pocket of Topoisomerase IV, it linked to the four key amino acids **Asn1042**, **Asp1069**, **Asp1077** and **Arg1132** via hydrogen bonding (Figure 3).

**Figure 4:**
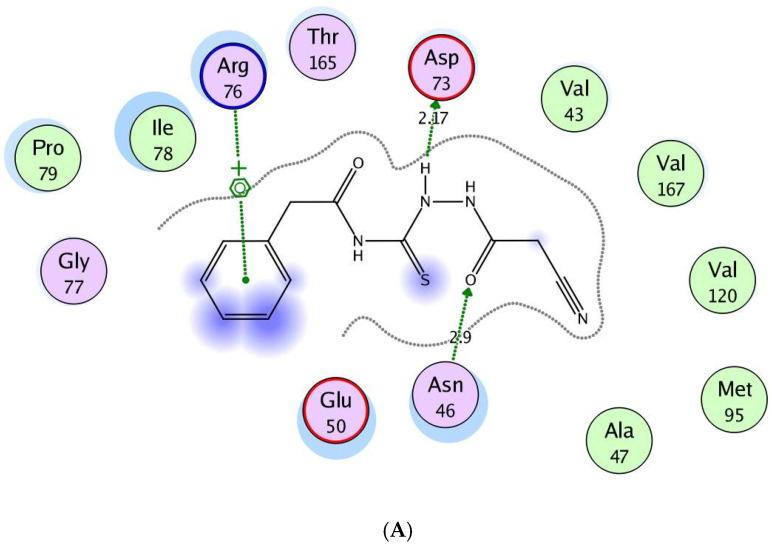

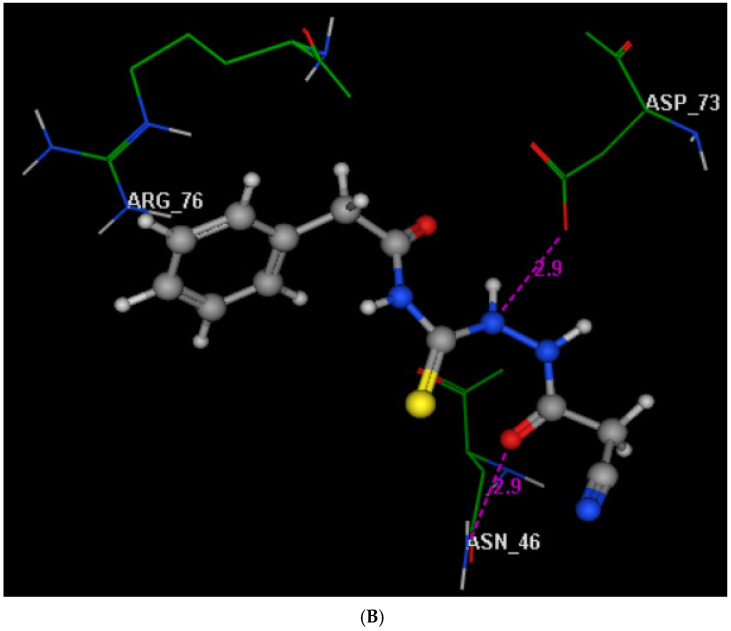
Two-dimensional (**A**) and three-dimensional (**B**) interaction diagrams of compound **8** docked in the active site of *E. coli* DNA gyrase B (PDB ID: 1AJ6) using MOE software. H-bond interactions with the protein are shown as dashed lines.

**Figure 5:**
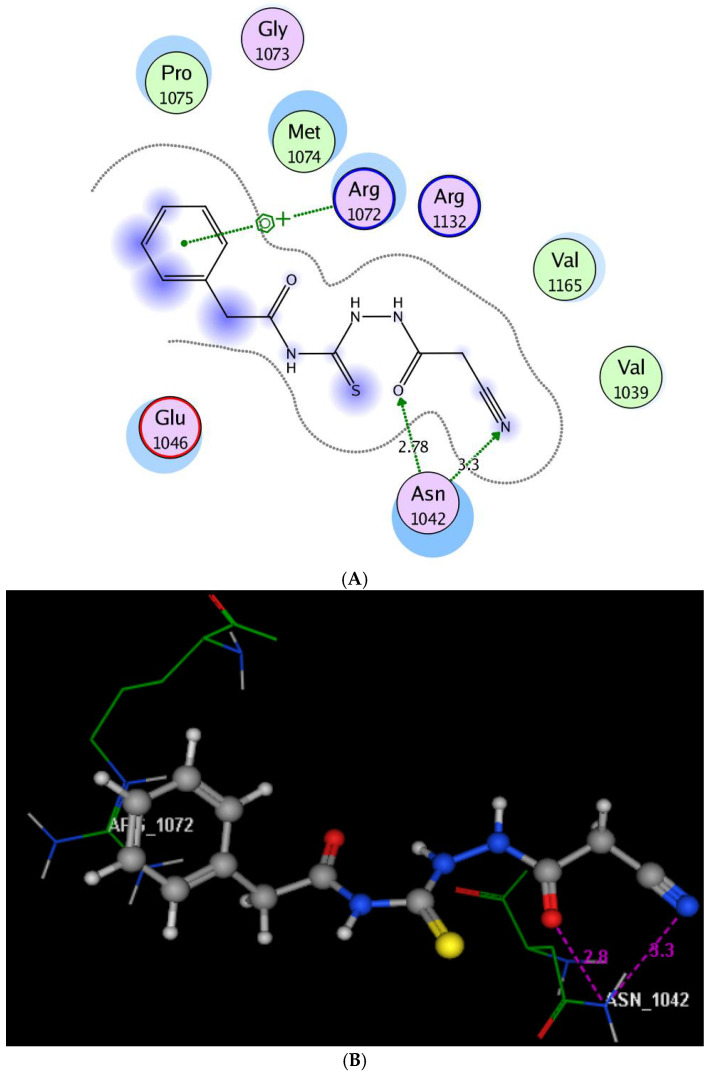
Two-dimensional (**A**) and three-dimensional (**B**) interaction diagrams of compound **8** docked in the active site of *E. coli* Topoisomerase IV (PDB ID: 1S14) using MOE software. H-bond interactions with the protein are shown as dashed lines.

**Figure 6:**
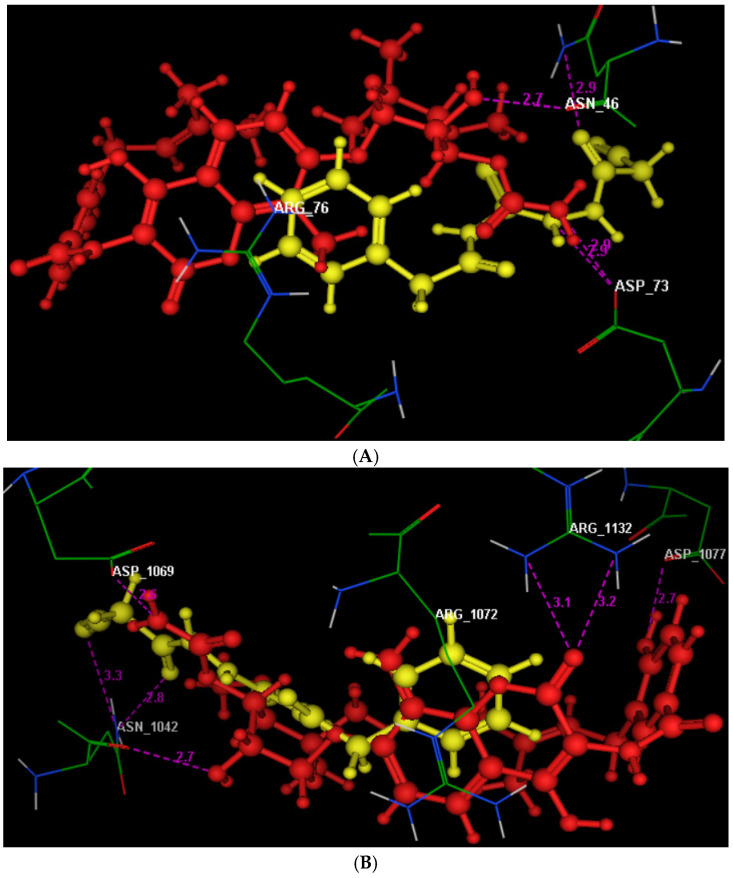
Three-dimensional representation of the docked novobiocin (red) in superimposition with compound **8** (yellow) in the active sites of *E. coli* DNA gyrase B (PDB ID: 1AJ6) (**A**) and Topoisomerase IV (PDB ID: 1S14) (**B**).

Then, docking study was performed for the most potent compound **8** to shed light on its potential binding modes and investigate its similarity to the native ligand, novobiocin. According to the docking simulation result observed in Figure 4 and Figure 5, compound **8** was embedded nicely within the active pocket of *E. coli* DNA gyrase B with binding energy of −12.23 kcal/mole and exactly with the same manner of novobiocin that was illustrated through superimposition between them (Figure 6a). The NH of thiourea moiety allowed hydrogen bond donor with the sidechain of **Asp73** and the oxygen of the cyanoacetyl group shared the fixation within the binding pocket through H-bond acceptor with the sidechain of **Asn46** (distance: 2.17 and 2.90 Å, respectively). Moreover, the similar arene–cation interaction with **Arg76** was noticed with the centroid of phenyl ring (Figure 4).

Regarding to the docking model within the active site of *E. coli* Topoisomerase IV, compound **8** resembles novobiocin in binding with only one amino acid **Asn1042** that was confirmed by superimposition in Figure 6b. The nitrogen and oxygen atoms of cyanoacetyl fragment formed two hydrogen bond acceptors with the sidechain of **Asn1042** (distance: 3.30 and 2.78 Å, respectively). Furthermore, the phenyl ring also took part in the hydrophobic interaction with **Arg1072** through an arene–cation interaction (Figure 5).

Based on the biological evaluations and molecular docking study we could deduce the following features in the most active compound **8**; thiosemicarbazide fragment connected to the cyano group via the acetamide moiety allowed for the formation of similar interactions (hydrogen bond acceptor, donor and arene–cation interaction) with the essential amino acids **Asn46**, **Asp73** and **Arg76** in the binding pocket of *E. coli* DNA gyrase B as novobiocin that was responsible for its superior antimicrobial activity through inhibition of *E. coli* DNA gyrase B (IC_50_ = 0.33 ± 1.25 µM). On the other hand, the key **Asn1042** played a crucial role in stability of compound **8** like novobiocin within the binding pocket of Topoisomerase IV through two hydrogen bond acceptors to exert good inhibitory activity (IC_50_ values 19.72 ± 1.00 µM). Furthermore, concerning the previous results, our derivatives could possibly target other receptors than DNA gyrase and topoisomerase IV. This point will be discussed and screened more in the future work.

## 3. Experimental

### 3.1. Chemistry

Melting points are uncorrected and measured on a Gallenkamp electric melting point apparatus. Infrared spectra carried out using potassium bromide disks on a FTIR Thermo Electron Nicolet 7600 (USA) infrared spectrometer at the central laboratory of faculty of science Ain shams University. ^1^HNMR spectra run at 300 MHz on a GEMINI 300 BB NMR spectrometer using tetramethylsilane (TMS) as internal standard in deuterated dimethylsulphoxide (DMSO-d_6_) at the main defense chemical laboratory. The mass spectra operating at 70 eV on Shimadzu GCMS-QP-1000EX mass spectrometer at the Regional center for Mycology and Biotechnology of Al-Azhar University. The elemental analyses performed on a Perkin-Elemer 2400 CHN elemental analyzer at the Micro analytical center of Cairo University. Antimicrobial activity was studied at Pharmacology Department, Faculty of Pharmacy, Mansoura University. N-Amino-4-benzylidene-2-phenyl-1*H*-imidazol-5(4*H*)-one **4** and 3-aryl-2-(3-phenylureido) acrylohydrazide **5a,b** were synthesized according to the method outlined in the literature [27,28].

#### 3.1.1. 3-(2-Phenylacetyl)-2-thioxoimidazolidin-4-one (**3**)

A solution of phenyl acetyl isothiocyanate **1** (0.01 mol) in dry acetonitrile (20 mL) and drops of pyridine was refluxed with ethyl glycinate hydrochloride (0.01 mol) for 4 h. The solvent was vacuum distilled and the residue was treated with cold ice. A solid product was obtained, filtered off and recrystallized from ethanol to give **3.** Yield 75%; colorless needles; m.p. 70–72 °C; IR (KBr) (υ, cm^−1^): 3245 (NH), 3071 (CH_arom_), 2994, 2940, 2908 (CH _alkyl_), 1741, 1642 (C=O), 1196 (C=S); ^1^HNMR (DMSO-*d*_6_) δ: 3.45 (s, 2H, Ph-CH_2_), 3.74 (AB quartet, 2H, CH_2_, *J* = 12Hz), 7.17–7.29 (m, 5H, Ar-H), 8.33 (br.s, 1H, NH, exchangeable with D_2_O); ^13^C-NMR (DMSO-*d*_6_) δ: 41.2 (ph-CH_2_), 42.9 (NH-CH_2_), 126.8, 128.6, 129.5, 136.7 (Ar-C), 171 (C=O), 171.8 (C=S); MS (70 eV) m/z (%): 234 (M^+.^, 23), 192 (9), 177 (11), 161 (14), 119 (18), 91 (16), 77 (25), 69 (92), 43 (100); Anal. calcd for C_11_H_10_N_2_O_2_S (234.27): C, 56.39; H, 4.30; N, 11.95. Found: C, 56.18; H, 3.93; N, 11.59.

#### 3.1.2. Ethyl ((2-phenylacetyl) carbono thionyl)glycinate (**2**)

To a solution of compound **3** (1 g) in ethanol (25 mL), hydrochloric acid (5 mL, 5 M) was added. The reaction mixture was refluxed for 1 h, after cooling the colorless solid product was obtained, filtered off, and recrystallized from ethanol to give compound **2**. Yield 92%; m.p. 120–122 °C; IR (KBr) (υ, cm^−1^): 3384 (NH), 3062, 3032 (CH_arom_), 2933, 22858, 2743 (CH _alkyl_), 1721, 1613 (C=O); ^1^HNMR (DMSO-*d*_6_) δ: 1.15 (t, 3H, CH_3_CH_2_O, *J* = 5.1, 5.4 Hz), 3.46 (s, 2H, PhCH_2_-), 3.79 (s, 2H, NH-CH_2_-), 4.08 (q, 2H, CH_3_CH_2_O, *J* = 5.1, 5,7 Hz), 7.22 (br.s, 1H, NH-CH_2_, exchangeable with D_2_O), 7.19–7.29 (m, 5H, Ar-H), 8.45 (br.s, 1H, CONHCS, exchangeable with D_2_O); ^13^C-NMR (DMSO-*d*_6_) δ: 14.5 (CH_3_), 41.3 (ph-CH_2_), 42.6 (NH-CH_2_), 60.9 (CH_2_), 126.8, 128.7, 129.5, 136.6 (Ar-C), 170.4 (C=O), 171.2 (C=S); MS (70 eV) m/z (%): 280 (M^+.^, 26), 263 (100), 207 (17), 193 (33), 178 (22), 134 (50), 103 (32), 91 (90); Anal. calcd for C_13_H_16_N_2_O_3_S (280.34): C, 55.67; H, 5.75; N, 9.99. Found: C, 55.28; H, 5.39; N, 9.71.

#### 3.1.3. Reactions of Isothiocyanate (**1**) with Different Amines. Synthesis of Compounds **6**, **7** and **8**

A mixture of compound **1** (0.01 mol) and N-aminoimidazole **4** (0.01 mol), carbohydrazide **5a,b** (0.01 mol) or cyanoacetohydrazide (0.01 mol) in dry acetonitrile (20 mL) was stirred at room temperature for 2–4 h. The precipitated solid was collected by filtration, dried and crystallized from ethanol to give the corresponding compounds **6**, **7a**, **7b** and **8**, respectively.

#### 3.1.4. 1-(4-Benzylidene-4,5-dihydro-5-oxo-2-phenylimidazol-1-yl)-3-(2-phenylacetyl)thiourea (**6**)

Yellow crystals; yield (90%); m.p. 238–240 °C (EtOH); IR (KBr) (υ, cm^−1^): 3191 (NH), 3059, 3027 (CH_arom_), 2928 (CH _alkyl_), 1701, 1640 (C=O), 1598 (C=N), 1163 (C=S); ^1^HNMR (DMSO-d_6_) δ: 3.68 (s, 2H, CH_2_), 7.25–8.32 (m, 16H, Ar-H+ CH=), 11.96 (br.s, 1H, CSNHCO, exchangeable with D_2_O), 12.31 (br.s, 1H, CSNHN-, exchangeable with D_2_O); ^13^C-NMR (DMSO-d_6_) δ: 42.9 (CH_2_), 127.5 (CH=C), 128.7–129.9 (Ar-C), 131.3, 134.3, 134.6, 136.6 (Ar-C), 133 (C=C), 160.6 (C=N), 167.3 (N-C=O), 172.2 (NH-C=O), 182.4 (C=S); MS (70 eV) m/z (%): 440 (M^+.^, 62), 321 (2), 119 (5), 105 (27), 91 (100), 77 (28), 59 (4); Anal. calcd for C_25_H_20_N_4_O_2_S (440.51): C, 68.16; H, 4.57; N, 12.71. Found: C, 67.76; H, 4.23; N, 12.46.

#### 3.1.5. 1-(2-Benzamido-3-phenylacryloyl)-4-phenylacetylthiosemicarbazide (**7a**)

White crystals; yield (92%); m.p. 190–192 °C; IR (KBr) (υ, cm^−1^): 3247, 3182 (NH), 3061, 3026 (CH_arom_), 1694, 1642 (C=O), 1599 (C=N), 1153 (C=S); ^1^HNMR (DMSO-*d*_6_) δ: 3.80 (s, 2H, CH_2_), 7.29–8.01 (m, 16H, Ar-H+ CH=), 10.15 (br.s, 1H, NHCOPh, exchangeable with D_2_O), 10.84 (br.s, 1H, CSNHNH, exchangeable with D_2_O), 11.88 (br.s, 1H, CSNHNH, exchangeable with D_2_O), 12.36 (br.s, 1H, CONHCS, exchangeable with D_2_O); ^13^C-NMR (DMSO-*d*_6_) δ: 42.7 (CH_2_), 127.5, 128.3 (C=C), 128.3-134.7 (Ar-C), 163, 166.6, 173.4 (C=O),178.1 (C=S); MS (70 eV) m/z (%):458 (M^+.^, 7), 353 (2), 265 (6), 237 (4), 208 (2), 134 (2), 119 (12), 105 (62), 91 (49), 77 (100), 64 (10); Anal. calcd for C_25_H_22_N_4_O_3_S (458.53): C, 65.48; H, 4.83; N, 12.21. Found: C, 65.12; H, 4.44; N, 11.89.

#### 3.1.6. 1-(2-Benzamido-3-(thiophen-2-yl)acryloyl)-4-phenylacetylthiosemicarbazide (**7b**)

White crystals; yield (85%); m.p. 212–214 °C; IR (KBr) (υ, cm^−1^): 3268, 3172 (NH), 3078, 3027 (CH_arom_), 1694, 1664, 1643 (C=O), 1617 (C=N), 1156 (C=S); ^1^HNMR (DMSO-*d*_6_) δ: 3.76 (s, 2H, CH_2_), 7.11–8.05 (m, 14H, Ar-H+ CH=), 9.95 (br.s, 1H, NHCOPh, exchangeable with D_2_O), 10.65 (br.s, 1H, CSNHNH, exchangeable with D_2_O), 11.84 (br.s, 1H, CSNHNH, exchangeable with D_2_O), 12.44 (br.s, 1H, CONHCS, exchangeable with D_2_O); ^13^C-NMR (DMSO-*d*_6_) δ: 42.6 (CH_2_), 124.5 (CH=C), 127.5–133.9 (Ar-C), 134 (NH-C=C), 134.7 (CH_2_-C=C), 131.8, 136.6 (S-C=C), 162.1, 166.8, 173.4 (C=O), 177 (C=S); MS (70 eV) m/z (%): 464 (M^+.^, 65), 357 (49), 345 (22), 256 (10), 236 (4), 134 (13), 120 (15),104 (29), 91 (4), 83 (7), 77 (48), 73 (72); Anal. calcd for C_23_H_20_N_4_O_3_S_2_ (464.55): C, 59.46; H, 4.33; N, 12.06. Found: C, 59.11; H, 3.95; N, 11.82.

#### 3.1.7. 1-(2-Cyanoacetyl)-4-phenylacetylthiosemicarbazide (**8**)

White crystals; yield (96%); m.p. 158–160 °C; IR (KBr) (υ, cm^−1^): 3282, 3194 (NH), 3012 (CH_arom_), 2933, 2911 (CH _alkyl_), 1694 (C=O), 1590 (C=N), 1150 (C=S); ^1^HNMR (DMSO-*d*_6_) δ: 3.72 (s, 2H, CH_2_Ph), 3.79 (s, 2H, CH_2_CN), 7.25–7.33 (m, 5H, Ar-H), 11.94 (br.s, 1H, CSNHNHCO, exchangeable with D2O), 11.77 (br.s, 1H, CSNHNHCO, exchangeable with D2O), 12.13(br.s, 1H, CONHCS, exchangeable with D2O); ^13^C-NMR (DMSO-*d*_6_) δ: 31.7 (CH_2_-CN) 42.5 (Ph-CH_2_), 115.8 (CN), 127.4–136.3 (Ar-C), 166.1, 166.7 (C=O), 172.9 (C=S); MS (70 eV) m/z (%): 276 (M^+.^, 16), 117 (66), 96 (2), 77 (19), 63 (100); Anal. calcd for C_12_H_12_N_4_O_2_S (276.31): C, 52.16; H, 4.37; N, 20.27. Found: C, 51.76; H, 4.09; N, 19.91.

#### 3.1.8. Synthesis of Compounds **9** and **10**

A solution of **8** (0.5 g) and catalytic amount of sodium hydroxide or hydrochloric acid (5 mL, 3 M) in ethanol (20 mL) was refluxed for 2 h. After cooling to room temperature, the reaction mixture was poured into ice, and then acidified with hydrochloric acid (1N) “in case NaOH”. The obtained precipitate was filtered off and recrystallized from ethanol to give the corresponding compounds **9** and **10**, respectively. 

##### 2-Benzyl-3,4-dihydro-4-thioxopyrazolo[1,5-*a*][1,3,5]triazin-7(6*H*)-one (**9**)

White powder; yield (81%); m.p. < 300 °C; IR (KBr) (υ, cm^−1^): 3161, 3106 (NH), 3013 (CH_arom_), 2854 (CH _alkyl_), 1620 (C=O), 1600 (C=N), 1173 (C=S); ^1^HNMR (DMSO-*d*_6_) δ: 3.93 (s, 2H, CH_2_Ph), 5.86 (s, 1H, CH=), 7.20–7.34 (m, 5H, Ar-H), 11.69 (br.s, 1H, NHCS, exchangeable with D_2_O), 13.65 (br.s, 1H, NHCO, exchangeable with D_2_O); ^13^C-NMR (DMSO-*d*_6_) δ: 40.6 (CH_2_) 86.1 (C=C-CO), 127, 128.7, 129.6, 136.2 (Ar-C), 145.9 (C=N), 154.1 (N-C=C), 167.2 (C=O), 168.1 (C=S); MS (70 eV) m/z (%): 258 (M^+.^, 68), 167 (2), 201 (5), 199 (8), 91 (100), 82 (2), 77 (10), 57 (2); Anal. calcd for C_12_H_10_N_4_OS (258.29): C, 55.80; H, 3.90; N, 21.69. Found: C, 55.43; H, 3.65; N, 21.30.

##### N-(5-(Cyanomethyl)-1,3,4-thiadiazol-2-yl)-2-phenylacetamide (**10**)

Colorless crystals; yield (58%); m.p. 198–200 °C; IR (KBr) (υ, cm^−1^): 3148 (NH), 3032 (CH_arom_), 2945, 2886, 2794 (CH _alkyl_), 2252 (CN), 1681 (C=O), 1558 (C=N); ^1^HNMR (DMSO-*d*_6_) δ: 3.81 (s, 2H, CH_2_Ph), 4.53 (s, 2H, CH_2_CN), 7.22–7.33 (m, 5H, Ar-H), 12.87 (br.s, 1H, NHCO, exchangeable with D2O); ^13^C-NMR (DMSO-*d*_6_) δ: 18.8 (CH_2_-CN), 41.9 (CH_2_), 117 (CN), 127.4, 128.9, 129.8, 134.9 (Ar-C), 154.4, 160.2 (S-C=N), 170.2 (C=O); MS (70 eV) m/z (%): 258 (M^+.^, 9), 118 (17), 91 (100), 65 (10); Anal. calcd for C_12_H_10_N_4_OS (258.29): C, 55.80; H, 3.90; N, 21.69. Found: C, 55.46; H, 3.54; N, 21.41.

#### 3.1.9. N-(5-(2-Imino-2H-chromen-3-yl)-1,3,4-thiadiazol-2-yl)-2-phenylacetamide (**11**)

To a mixture of compound **10** (0.01 mol), and salicylaldehyde (0.01 mol) in ethyl alcohol (30 mL), ammonium acetate (0.01 mol) was added, and then heated under reflux for 6 h. The formed solid product was filtered off, dried and recrystallized from dimethylformamide to give compound **11**. Pale yellow needles; yield (94%); m.p. < 250 °C; IR (KBr) (υ, cm^−1^): 3288 (NH), 3024 (CH_arom_), 2925, 2856, 2725 (CH _alkyl_), 1692 (C=O), 1659, 16047 (C=N); ^1^HNMR (DMSO-*d*_6_) δ: 3.84 (s, 2H, CH_2_Ph), 7.20–7.74 (m, 9H, Ar-H), 8.57 (s, 1H, CH=), 9.01 (br.s, 1H, NH=, exchangeable with D_2_O), 12.71 (br.s, 1H, NHCO, exchangeable with D_2_O); ^13^C-NMR (DMSO-*d*_6_) δ: 42.1 (CH_2_), 115.5–135.2 (Ar-C), 153.8 (O-C=C), 153.4, 155.8 (S-C=N), 162.4 (C=NH), 169.9 (C=O); MS (70 eV) m/z (%): 363 (M^+.^, 6), 347 (1), 245 (2), 101 (6), 91 (100), 77 (5), 44 (2); Anal. calcd for C_19_H_13_N_3_O_3_S (363.38): C, 62.80; H, 3.60; N, 11.56. Found: C, 62.45; H, 3.31; N, 11.16.

#### 3.1.10. 5-Benzylidene-5,6-dihydro-N-(1-hydroxy-2-phenylethylidene)-6-oxo-3-phenyl-1,2,4-triazine-2(1*H*)-carbothioamide (**12**)

A solution of compound **7a** (0.01 mol) in alcoholic sodium hydroxide (2 gm NaOH in 20 mL ethanol, 10%) was refluxed for 3h. The reaction mixture was cooled to room temperature, and then poured into ice/HCl. The formed solid product was filtered off and recrystallized from benzene to give compound **12**. Pale brown crystals; yield (65%); m.p. 138–140 °C; IR (KBr) (υ, cm^−1^): 3240 (NH), 3034 (CH_arom_), 2924, 2855 (CH _alkyl_), 1648 (C=O) 1602 (C=N); ^1^HNMR (DMSO-*d*_6_) δ: 3.85 (s, 2H, CH_2_Ph), 7.05–7.97 (m, 16H, Ar-H+ CH=), 10.12 (br.s, 1H, NNH, exchangeable with D_2_O), 13.52 (br.s, 1H, CSNHCO, exchangeable with D_2_O), 13.64 (br.s, 1H, OHC=N, exchangeable with D_2_O); ^13^C-NMR (DMSO-*d*_6_) δ: 41.5, 42.2 (CH_2_), 114.6, 134.1 (C=C), 127.9–137.4 (Ar-C), 158.3 (C=N), 163, 165.8, 172.7 (C=O), 171.3 (C-OH), 183.8, 188.2 (C=S); MS (70 eV) m/z (%): 440 (M^+.^, 10), 175 (23), 144 (12), 105 (93), 71 (100); Anal. calcd for C_25_H_20_N_4_O_2_S (440.51): C, 68.16; H, 4.57; N, 12.71. Found: C, 67.79; H, 4.29; N, 12.31.

#### 3.1.11. N-(1-(5-(2-Phenylacetamido)-1,3,4-thiadiazol-2-yl)-2-(thiophen-2-yl)vinyl)benzamide (**13**)

A solution of compound **7b** (0.15 gm) in POCl_3_ (10 mL) was heated under reflux for 2 h. The reaction mixture was cooled at room temperature and poured into ice water. The obtained solid was filtered off and recrystallized from ethanol/water to give compound **13**. Green crystals; yield (98%); m.p. 110–112 °C; IR (KBr) (υ, cm^−1^): 3120 (NH), 3036 (CH_arom_), 2925, 2853 (CH _alkyl_), 1694, 1641 (C=O); ^1^HNMR (DMSO-*d*_6_) δ: 3.80 (s, 2H, CH_2_Ph), 7.10–8.05 (m, 14H, Ar-H+ CH=), 9.73 (br.s, 1H, NHCOPh, exchangeable with D_2_O), 10.31 (br.s, 1H, NHCOCH_2_Ph, exchangeable with D_2_O); ^13^C-NMR (DMSO-*d*_6_) δ: 41 (CH_2_), 123.6 (CH=C), 127.1-136.9 (Ar-C), 137.3 (NH-C=C), 158.6, 163.1 (S-C=N), 166.8, 170 (C=O); MS (70 eV) m/z (%): 446 (M^+.^, 12), 327 (23), 230 (10), 105 (93), 91 (20), 65 (100), 44 (18); Anal. calcd for C_23_H_18_N_4_O_2_S_2_ (446.54): C, 61.86; H, 4.06; N, 12.54. Found: C, 61.46; H, 3.77; N, 12.18.

### 3.2. Biological Activity

#### 3.2.1. Antimicrobial Sensitivity Assay

The antimicrobial assay was performed in vitro for the target compounds **2**–**13** against *Staphylococcus aureus* ATCC 29213, *Bacillus subtilis* ATCC 6633 as Gram-positive bacteria, *Escherichia coli* ATCC 25922, *Pseudomonas aeruginosa* ATCC 27853 as Gram-negative bacteria and *Candida albicans* ATCC 10231, *Aspergillus flavus* ATCC 46283 as fungi. At the first, 100 μL of the test bacteria/fungi were grown in 10 mL of fresh media until they reached a count of approximately 10^8^ cells/ml for bacteria or 10^5^ cells/mL for fungi. One milliliter of each sample (at 0.5 mg/mL) was added to each well (10 mm diameter holes cut in the agar gel). The plates were incubated for 24 h at 37 °C (for bacteria and yeast) and for 72 h at 27 °C (for filamentous fungi), each test was repeated three times. After incubation, the microorganism’s growth was observed. Ciprofloxacin and clotrimazole were used as standard antibacterial and antifungal drugs, respectively. The resulting inhibition zone diameters were measured in millimeters using the diffusion technique [24]. The active compounds **6**, **7a**, **7b**, **8**, **9, 12** and **13** were further investigated to determine their antimicrobial activity expressed in terms of minimum inhibitory concentration (MIC) using the modified agar well diffusion method that mentioned above. The different concentrations (triplicate) of each compound were tested and compared with standard drugs. 

#### 3.2.2. MTT Assay for Cytotoxic Activity

The cytotoxic activities of the highly potent derivatives as antimicrobials **6**, **7a**, **7b**, **8**, **9**, **12** and **13** were assessed against both human breast cancer (MCF-7) and normal kidney epithelial cell lines (Vero cells) that were obtained from Sigma-Aldrich Chemical Company, St. Louis, MO, USA. The experiment performed using MTT assay and cisplatin as a standard following the previously mentioned techniques [24].

#### 3.2.3. Kinase Inhibition Assay 

The in vitro enzyme inhibition assessment for the most active derivative **8** was carried out in the confirmatory diagnostic unit, Vacsera, Egypt. The screening performed against *E. coli* DNA gyrase B, *E. coli* Topoisomerase IV and dihydrofolate reductase enzymes. *E. coli* DNA gyrase microplate assay kit (Inspiralis), *E. coli* topoisomerase IV decatenation kit (Inspiralis) and dihydrofolate reductase inhibitor screening Kit (Colorimetric) Bio Vision have been used for the anticipated assay according to the optimized protocol by the manufacturer. The used reference drugs were novobiocin for *E. coli* DNA gyrase B and *E. coli* Topoisomerase IV and methotrexate for DHFR according to the previously mentioned methods [22,24,29]. The obtained data are depicted as IC_50_ values in Table 4.

### 3.3. Molecular Modeling Study

The 2D structure of the newly synthesized compound **8** was drawn by chem. Draw. Then, the protonated 3D was built using standard bond angles and lengths, with the MOE 10.2008 software [30], following geometry optimization and energy minimization were done to employ the Conf Search module in MOE, then the MOE file was saved to be available for the docking process. From the protein data bank, the co-crystallized structures of *E. coli* DNA gyrase B and Topoisomerase IV with their ligand novobiocin were downloaded (PDB code: 1AJ6 and 1S14), respectively [26,31]. All minimizations were performed with MOE until an RMSD gradient of 0.05 kcal∙mol^−1^Å^−1^ with the MMFF94x force field and the partial charges were automatically calculated. The structures of the two enzymes were prepared for molecular docking using the Protonate 3D protocol in MOE with the default options. Triangle Matcher placement method and London dG scoring function were applied in the docking protocol. Firstly, the validation processes were confirmed by docking of the original ligand, followed by docking of the compound **8** into the active sites after removing the co-crystallized ligand according to the reported procedure [26]. 

## 4. Conclusions

A series of novel thiourea derivatives **2**–**13** bearing different heterocyclic systems was synthesized and screened for their biological activities. Compound **8** showed significant antimicrobial activity against almost tested strains with inhibition zone diameters (in mm) ranging from 42 ± 0.30 to 18 ± 0.41 and MIC values ranged from 0.95 ± 0.22 to 3.25 ± 1.00 μg/mL comparing with the reference drugs. Furthermore, the cytotoxic screening against MCF-7 cancer cells compared to normal kidney epithelial cell lines (Vero cells) revealed the potential cytotoxic effects of the synthesized derivatives. Based on the promising in vitro inhibition results of compound **8** against *E. coli* DNA B gyrase and Topoisomerase IV, the thiosemicarbazide derivative **8** bearing cyano group via acetamide moiety illustrated good fitting and favorable binding interactions in the docking study in comparison with the native ligand, novobiocin.

## Figures and Tables

**Table 1:** Antimicrobial studies for the synthesized compounds **2**–**13** at 1 µg/mL measured as inhibition zone diameter (mm) by well diffusion agar assay.

Compd.	Mean Diameter of Inhibition Zone (Mean ± SEM) (mm)					
	Gram Positive Bacteria		Gram Negative Bacteria		Fungi	
	*S. aureus* ATCC 29213	*B. subtilis* ATCC 6633	*P. aeruginosa* ATCC 27853	*E. coli* ATCC 25922	*A. flavus* ATCC 46283	*C. albicans* ATCC 10231
**2**	9 ± 0.61	NA	NA	NA	NA	NA
**3**	NA	NA	NA	NA	NA	NA
**6**	27 ± 0.20	30 ± 0.26	21 ± 0.26	12 ± 0.10	9 ± 0.03	6 ± 0.22
**7a**	30 ± 0.22	33 ± 0.50	27 ± 0.03	18 ± 0.22	24 ± 0.02	15 ± 0.30
**7b**	33 ± 0.01	35 ± 0.26	26 ± 0.21	18 ± 0.41	25 ± 0.15	16 ± 0.12
**8**	39 ± 1.21	42 ± 0.30	36 ± 0.12	27 ± 0.62	27 ± 0.21	18 ± 0.05
**9**	21 ± 0.30	18 ± 0.25	9 ± 0.25	NA	15 ± 0.33	9 ± 0.20
**10**	15 ± 0.42	12 ± 0.21	6 ± 0.02	NA	6 ± 0.41	NA
**11**	12 ± 0.30	6 ± 0.53	NA	NA	NA	NA
**12**	22 ± 0.05	23 ± 0.01	17 ± 0.20	5 ± 0.58	18 ± 0.03	14 ± 0.21
**13**	21 ± 0.10	24 ± 1.11	15 ± 0.11	6 ± 0.15	18 ± 0.22	12 ± 0.46
**Ciprofloxacin**	24 ± 0.60	23 ± 0.20	23 ± 0.90	26 ± 0.25	NA	NA
**Clotrimazole**	NA	NA	NA	NA	25 ± 0.40	27 ± 0.21

NA: No Activity, Ciprofloxacin and Clotrimazole were used as antibacterial and antifungal standards, respectively, SEM = standard error mean; each value is the mean of three measures.

**Table 2:** Minimum inhibitory concentration (MIC; μg/mL) of the most active derivatives.

Compd.	MIC (Mean ± SEM) (μg/mL)					
	Gram Positive Bacteria		Gram Negative Bacteria		Fungi	
	*S. aureus* ATCC 29213	*B. subtilis* ATCC 6633	*P. aeruginosa* ATCC 27853	*E. coli* ATCC 25922	*A. flavus* ATCC 46283	*C. albicans* ATCC 10231
**6**	5.12 ± 0.05	2.29 ± 0.05	2.48 ± 0.11	7.80 ± 0.10	26.20 ± 0.03	91 ± 0.02
**7a**	4.15 ± 0.22	1.39 ± 0.50	2.81 ± 0.01	4.22 ± 0.12	5.21 ± 0.11	64 ± 0.41
**7b**	3.90 ± 0.26	1.46 ± 0.10	2.72 ± 0.15	4.68 ± 0.13	5.70 ± 0.01	76 ± 0.31
**8**	3.25 ± 1.00	1.38 ± 0.25	1.25 ± 0.50	0.95 ± 0.22	2.11 ± 0.51	39.8 ± 0.20
**9**	9.92 ± 0.30	8.60 ± 0.25	17.62 ± 0.20	-	20.45 ± 0.30	94 ± 0.10
**12**	5.22 ± 0.03	2.30 ± 0.05	12.50 ± 0.20	27 ± 0.02	15.60 ± 0.01	85 ± 0.15
**13**	5.60 ± 0.15	2.72 ± 1.11	13.41 ± 0.10	33. ± 0.10	14.55 ± 0.20	83 ± 0.22
**Ciprofloxacin**	5.85 ± 0.13	2.90 ± 0.02	2.90 ± 0.04	2.90 ± 0.25	-	-
**Clotrimazole**	-	-	-	-	4.25 ± 0.05	12.5 ± 0.15

-: Not tested, SEM = mean of the standard error; each value is the mean of three values.

**Table 3:** Cytotoxic activity for the highly potent synthesized compounds against breast cancer (MCF-7) and normal kidney epithelial cell line (Vero cells).

Compd. No.	IC_50_ (Mean ± SEM) (µM) ^a^	IC_50_ (Mean ± SEM) (µM) ^a^
	MCF-7 Cells	Vero Cells
**Cisplatin**	8.897 ± 0.37	92.16 ± 0.07
**6**	23.69 ± 0.96	122.81 ± 0.40
**7a**	10.17 ± 0.65	149.10 ± 0.21
**7b**	11.589 ± 0.59	133.26 ± 0.40
**8**	22.35 ± 0.36	144.72 ± 0.36
**9**	26.45 ± 0.46	62.45 ± 0.20
**12**	35.92 ± 0.85	79.19 ± 0.28
**13**	37.68 ± 0.93	84.11 ± 0.32

^a^ IC_50_ values were calculated from the mean values of data from three separate experiments.

**Table 4:** Inhibitory assay of compound **8** against *Escherichia coli* DNA gyrase B, *E. coli* Topoisomerase IV and DHFR kinases.

Kinase	IC_50_ (mean ± SEM) (µM)		
	8	Novobiocin	Methotrexate
**DNA gyrase B**	0.33 ± 1.25	0.28 ± 1.45	-
**DNA topoisomerase IV**	19.72 ± 1.00	10.65 ± 1.02	-
**DHFR**	189.47 ± 1.06	-	0.14 ± 1.62

IC_50_: Compound concentration required to inhibit the enzyme activity by 50%, SEM = Standard error mean; each value is the mean of three values.
